# Rapid Responses to Reverse T_3_ Hormone in Immature Rat Sertoli Cells: Calcium Uptake and Exocytosis Mediated by Integrin

**DOI:** 10.1371/journal.pone.0077176

**Published:** 2013-10-10

**Authors:** Ana Paula Zanatta, Leila Zanatta, Renata Gonçalves, Ariane Zamoner, Fátima Regina Mena Barreto Silva

**Affiliations:** 1 Departamento de Bioquímica, Centro de Ciências Biológicas, Universidade Federal de Santa Catarina, Florianópolis-Santa Catarina, Brazil; 2 Universidade Comunitária da Região de Chapecó, Chapecó, Santa Catarina, Brazil; Massachusetts General Hospital, United States of America

## Abstract

There is increasing experimental evidence of the nongenomic action of thyroid hormones mediated by receptors located in the plasma membrane or inside cells. The aim of this work was to characterize the reverse T_3_ (rT_3_) action on calcium uptake and its involvement in immature rat Sertoli cell secretion. The results presented herein show that very low concentrations of rT_3_ are able to increase calcium uptake after 1 min of exposure. The implication of T-type voltage-dependent calcium channels and chloride channels in the effect of rT_3_ was evidenced using flunarizine and 9-anthracene, respectively. Also, the rT_3_-induced calcium uptake was blocked in the presence of the RGD peptide (an inhibitor of integrin-ligand interactions). Therefore, our findings suggest that calcium uptake stimulated by rT_3_ may be mediated by integrin α_v_β_3_. In addition, it was demonstrated that calcium uptake stimulated by rT_3_ is PKC and ERK-dependent. Furthermore, the outcomes indicate that rT_3_ also stimulates cellular secretion since the cells manifested a loss of fluorescence after 4 min incubation, indicating an exocytic quinacrine release that seems to be mediated by the integrin receptor. These findings indicate that rT_3_ modulates the calcium entry and cellular secretion, which might play a role in the regulation of a plethora of intracellular processes involved in male reproductive physiology.

## Introduction

Thyroid hormones (THs) are iodinated compounds known to inﬂuence gene expression in virtually every vertebrate cell. THs action is critically important for development, tissue differentiation, and maintenance of metabolic balance in mammals. Thyroxine (3,5,3’,5’-L-tetraiodothyronine; T_4_) is known to be the main secretory product of the thyroid gland in all vertebrates, and can be activated to triiodothyronine (3,5,3’-triiodothyronine; T_3_) in a stage- and tissue-speciﬁc manner by phenolic ring deiodination (outer ring deiodination) catalyzed by two iodothyronine deiodinases, D1 and D2. A third deiodinase, D3, promotes deiodination at the tyrosyl ring producing reverse T_3_ (3,3’,5’-triiodothyronine; rT_3_) and T_2_ (3,3’-diiodothyronine) [1,2]. All three deiodinases, D1, D2, and D3, are expressed in testis at different levels from weanling to adult life, however, D3 activity predominates in the developmental period and then declines in adult life [3].

THs are important modulators of spermatogenesis and steroidogenesis in the testis. The presence of specific nuclear thyroid hormone receptors (TRs), described in prepubertal Sertoli cells, implies the existence of an early and critical influence of thyroid hormones on testis development [4]. Accordingly, alterations in thyroid activity are frequently associated with changes in male reproductive functions, since hypothyroidism is associated with a marked delay in sexual maturation and development [5]. 

The classical mechanism of THs has been established as a genomic action, including binding to intracellular hormone receptors that share the characteristics of nuclear transcription and protein synthesis [6,7]. These events are characterized by a considerable latency with response times ranging from hours to days [7,8]. In general, THs modulate a large number of metabolic processes but not all of these actions are due to effects on nuclear transcription. 

Actions of THs that are independent of ligand binding to nuclear thyroid receptors are called rapid or nongenomic actions. This mechanism is independent of active protein synthesis, initiating in the plasma membrane [9]. It typically has a time-course of seconds or minutes and is frequently associated with secondary messenger and kinase signaling pathways [10]. 

Previous studies in our laboratory demonstrated some nongenomic effects in testes cells, including, amino acid accumulation [11-14], ion fluxes across plasma membrane [14,15], hyperpolarization of Sertoli cells [12,13], calcium influx [14,16-18], modulation of extracellular nucleotide levels [19] and alteration in the intermediate filament cytoskeleton dynamics [15]. In the other tissues, THs promote, through nongenomic actions, insertion of Na^+^,K^+^-ATPase into the plasma membrane, as well as the modulatory activity of this enzyme [20-22], intracellular shuttling of TRs resident in cytoplasm to the nucleus [23,24], and regulation of the state of the actin cytoskeleton [25] such as regulation of specific gene expression.

Secretory activities of Sertoli cells are critical to spermatogenesis [26]. Sertoli cells express a variety of ion channels involved in cellular secretory functions [27,28], and an increase in the intracellular calcium concentration ([Ca^2+^]i) is a key signal triggering exocytosis in these cells [29]. The role of cytosolic Ca^2+^ is directly involved in the fusion of the secretory vesicles with the plasma membrane (for review [30]), and in the several distinct maturation steps of these secretory vesicles prior to fusion [31]. Sertoli cells contribute to spermatogenesis since they supply the seminiferous epithelium with a rich ionic fluid and synthesize specific proteins, such as transferrin and androgen-binding protein, in combination with a series of other important factors that maintain ongoing and normal germ cell development [32,33].

Until recently, rT_3_ was regarded as an inactive hormone, however, studies in our group has demonstrated that this T_4_ metabolite stimulates amino acid accumulation (a specific plasma membrane transport system) in immature rat testis [34]. Furthermore, rT_3_ regulates actin polymerization [25] and the mobility of brain cells during brain development through nongenomic signaling [25,35]. In fact, the rT_3_ assay has been used in order to clarify the specificity of TH and is currently used in our laboratory [34]. We have previously showed that T_4_ effect was 10^6^ times more potent than T_3_ on amino acid accumulation [13]. Latter, we also demonstrated that T_4_ and T_3_ have particular specificity of action on calcium influx in cerebral cortex [56]. Also, in the testis, we showed a very fast and specific effect of T_4_ on calcium influx [14] that was not observed for T_3_. In addition, recently we find that rT_3_ and T_4_ have similar potency on amino acid accumulation, although rT_3_ to be significantly most efficient than T_4_, in immature rat testis to mediate plasma membrane rapid responses [34]. So, since these results clearly show that T_4_, T_3_ and rT_3_ specificity for rapid responses in testis or Sertoli cells are quite different, in this study we investigated the involvement of integrin on calcium uptake and exocytosis triggered by rT_3_ in immature rat Sertoli cells.

## Materials and Methods

### Materials

3,3’,5’-triiodothyronine (reverse T_3_, rT_3_; purity ≥ 97 %), Arg-Gly-Asp (RGD), 1,2-bis(2-aminophenoxy)ethane-N,N,N’,N’-tetraacetic acid tetrakis (acetoxymethyl ester) (BAPTA-AM), 9-anthracene carboxylic acid (9-AC), flunarizine, 2-(2-amino-3-methoxyphenyl)-4H-1-benzopyran-4-one (PD 98059), stearoylcarnitine, quinacrine, Dulbecco’s modified Eagle’s medium (DMEM), Ham’s F12 medium, penicillin, streptomycin, kanamycin and amphotericin B, Serum Replacement 3, bovine pancreas deoxyribonuclease (DNase type I), hyaluronidase (type I-S), trypsin, soybean trypsin inhibitor, sodium pyruvate, D-glucose, Hepes, and sodium bicarbonate were purchased from Sigma-Aldrich Chemical Co. (St. Louis, MO, USA). Collagenase-Dispase and bovine serum albumin (BSA) were acquired from Roche Diagnostics (Indianapolis, IN, USA). [^45^Ca]CaCl_2_ (sp. act. 321 KBq/mg Ca^2+^) and Optiphase Hisafe III biodegradable liquid scintillation were purchased from PerkinElmer (Boston, MA, USA). All other chemicals were of analytical grade.

### Animals

Male wistar rats (*Rattus norvegicus*) weighing ± 20 g from the Central Animal House-UFSC were bred in our animal house and maintained in an air-conditioned room (21 °C) with controlled lighting (12 h/12 h light/dark cycle). The suckling rats were kept with their mothers until sacrifice by decapitation. Pelleted food (Nuvital, Nuvilab CR1, Curitiba, PR, Brazil) and tap water were available *ad libitum*. All the animals were carefully monitored and maintained in accordance with ethical recommendations of The Brazilian Veterinary Medicine Council and the Brazilian College of Animal Experimentation. The protocol was approved by the Committee on the Ethics of Animal Experiments of the Federal University of Santa Catarina (Permit Number: CEUA/PP00418).

### Primary Culture of Sertoli Cells and Calcium Uptake

Sertoli cells were obtained from 11-day-old Wistar rats. Rats were killed by decapitation, and testes were removed and decapsulated. Sertoli cells were obtained by sequential enzymatic digestion as previously described by Dorrington et al. [36]. Sertoli cells were seeded at a concentration of 200,000 cells/cm^2^ in 24-well culture plates (Falcon, Deutscher, Brummath, France) and cultured for 72 h in Ham’s F12/DMEM (1:1) medium supplemented with Serum Replacement 3, 2.2 g/L sodium bicarbonate, antibiotics (50,000 IU/L penicillin, 50 mg/L streptomycin, and 50 mg/L kanamycin), and a fungicide (0.25 mg/L amphotericin B), in a humidified atmosphere of 5% CO_2_ and 95% air at 34 °C. Three days after being plated, residual germ cells were removed by a hypotonic treatment using 20 mM Tris-HCl (pH 7.2) for 150 s. [37]. Cells were washed with PBS, and fresh Ham’s F12/DMEM (1:1) medium was added. Five days after being plated, cells were preincubated in Krebs Ringer-bicarbonate buffer (KRb) (122 mM NaCl, 3 mM KCl, 1.2 mM MgSO_4_, 1.3 mM CaCl_2_, 0.4 mM KH_2_PO_4_, 25 mM NaHCO_3_ and glucose 5 mM) for 15 min in a Dubnoff metabolic incubator at 34 °C (pH 7.4) and gassed with an O_2_/ CO_2_ mixture (95:5, v/v). The medium was then replaced with fresh KRb containing 0.1 μCi/mL ^45^Ca^2+^ and left for 60 min. For calcium uptake measurements, cells were incubated for a further 30 s, 1 min or 5 min, in the absence (control) or presence of rT_3_ (from 10^-19^ to 10^-7^ M). The rT_3_ was dissolved in 0.01 M NaOH-saline (stock solution) to be further diluted to the final concentrations in KRb buffer. In some experiments, channel blockers or kinase inhibitors were added during the last 15 min before the hormone was added and maintained during the incubation period (see figure legends). The following drugs were used: BAPTA-AM (50 μM) (intracellular calcium chelator; [17]), 9-AC (1 μM) (blocker for calcium-dependent Cl^-^ channels; [18]), flunarizine (1 μM) (T-type voltage-dependent Ca^2+^ channel blocker; [18]), PD 98059 (30 μM) (MEK inhibitor; [18]), RGD peptide (500 nM) (TH binding on α_v_β_3_ receptor blocker; [38]) and stearoylcarnitine (1 μM) (PKC inhibitor; [39]).

Extracellular ^45^Ca^2+^ from primary Sertoli cells culture was thoroughly washed off in 127.5 mM NaCl, 4.6 mM KCl, 1.2 mM MgSO_4_, 10 mM HEPES, 11 mM glucose, and 10 mM LaCl_3_, at pH 7.4 (30 min in washing solution). The presence of La^3+^ during the washing stage was found to be essential to prevent release of the intracellular ^45^Ca^2+^ [40]. After La^3+^ tissue washing, cells were homogenized with 0.5 M NaOH solution; 100 μL aliquots were placed in scintillation fluid for counting in a Beckman coulter beta liquid scintillation spectrometer (model LS 6500; Fullerton, California, USA), and 50 µL aliquots were used for total protein quantification by the Lowry method [41]. The results were expressed as pmol ^45^Ca^2+^/μg of protein [16].

### Secretory activity of Sertoli cells

Sertoli cells were obtained from 11-day-old Wistar rats. On day 5 after plating, the cells were washed in Hank’s Buffered Salt Solution (HBSS) (136.9 mM NaCl, 16.7 mM NaHCO_3_, 1.3 mM CaCl_2_, 5.4 mM KCl, 0.65 mM MgSO_4,_ 0.27 mM Na_2_HPO_4_, 0.44 mM KH_2_PO_4_, 6.1 mM glucose). The medium was then replaced with fresh HBSS containing 3 µM quinacrine and cells were incubated for 30 min at 34 °C. The time-course of rT_3_ (10^-17^ M) was carried out at 1, 2, 3, 4, 5, 6, 7, 8, 9 and 10 min based on a similar approach previously used by our group [42]. When RGD and flunarizine were used, Sertoli cells were treated for 10 min with the drugs prior to incubation with quinacrine.

### Exocytosis imaging in primary culture of Sertoli cells

Microscopy imaging was performed on quinacrine-loaded live Sertoli cells as described by Menegaz et al. [42]. Briefly, cells were washed with HBSS and loaded with 3 µM quinacrine dissolved in HBSS for 30 min at 34 °C. Sertoli cells were viewed under an Olympus BX41fluorescence microscope using a FITC filter. Exocytosis was identified as the rapid loss of quinacrine fluorescence when released into the medium, indicating fusion of secretory vesicles with the plasma membrane with/without the hormone stimulus. Images were obtained with a QColor 3C digital camera (Q-imaging) at a scanning rate of 1 image/60 s and processed with Q-capture Pro 5.1 software program (Q-imaging).

### Statistical analysis

The results are means ± S.E.M. When multiple comparisons were performed, evaluation was carried out using one-way ANOVA followed by Bonferroni multiple comparison test or unpaired Student’s *t*-test was used to determine the significance of differences between groups. Differences were considered to be significant when *p* < 0.05.

## Results

### Rapid response of reverse T_3_ (rT_3_) in calcium uptake by Sertoli cells

**Figure 1:**
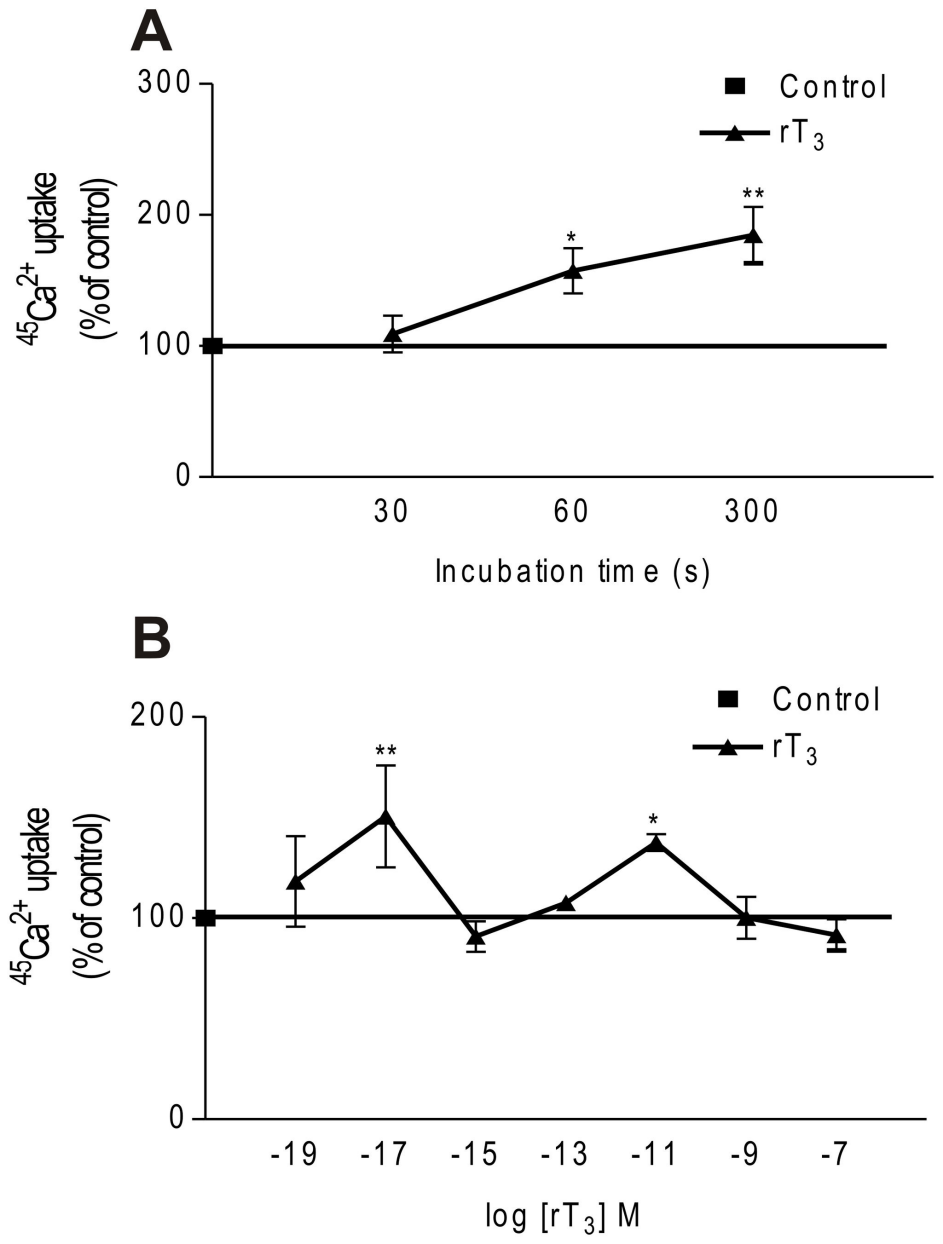
Time-course and dose-response curve of rT_3_ on Ca^2+^ uptake in Sertoli cells. (A) Time-course effect of rT_3_. Pre-incubation: 15 min in KRb, additional pre-incubation: 60 min with 0.1 µCi/mL of ^45^Ca^2+^ and incubation time: 30, 60 and 300 s with 0.1 µCi/mL of ^45^Ca^2+^ in the presence or absence of rT_3_ (10^-17^ M). Means ± S.E.M. n= 4 for all groups. **P < 0.01 and *p < 0.05 compared with control group. (B) Dose-response curve for rT_3_ in relation to Ca^2+^ uptake in Sertoli cells. Pre-incubation: 15 min in KRb, additional pre-incubation: 60 min with 0.1 µCi/mL of ^45^Ca^2+^ and incubation time: 60 s with 0.1 µCi/mL of ^45^Ca^2+^ in the presence or absence of rT_3_. Means ± S.E.M. For control and rT_3_ (10^-19^, 10^-17^, 10^-15^, 10^-13^, 10^-11^, 10^-9^ and 10^-7^ M), n=4 for each group. **P < 0.01 and *p < 0.05 compared with control group.

In this study we investigated the rapid action of rT_3_ in 11-day-old rat Sertoli cells using the radioisotope ^45^Ca^2+^, an accurate approach to measuring rapid effects on the plasma membrane. In the presence of 10^-17^ M rT_3,_ the calcium uptake increased from 30 s until the maximum period studied (5 min). At 60 and 300 s a significant stimulatory effect of the hormone on calcium uptake was observed compared to the control group at 30 s (Figure 1A). As can be observed, addition of 10^-17^ and 10^-11^ M rT_3_ to the cultures for 60 s caused a significant increase (50% and 37%, respectively) in calcium uptake by these cells compared with the control group. On the other hand, 10^-19^, 10^-15^, 10^-13^, 10^-9^ and 10^-7^ M rT_3_ did not caused any significant calcium uptake (Figure 1B). Since the aim of this study was to evaluate the rapid response of rT_3_ we applied 60 s and 10^-17^ M in subsequent experiments.

### Evidence for plasma membrane receptor mediation of rT_3_ stimulation of calcium uptake

**Figure 2:**
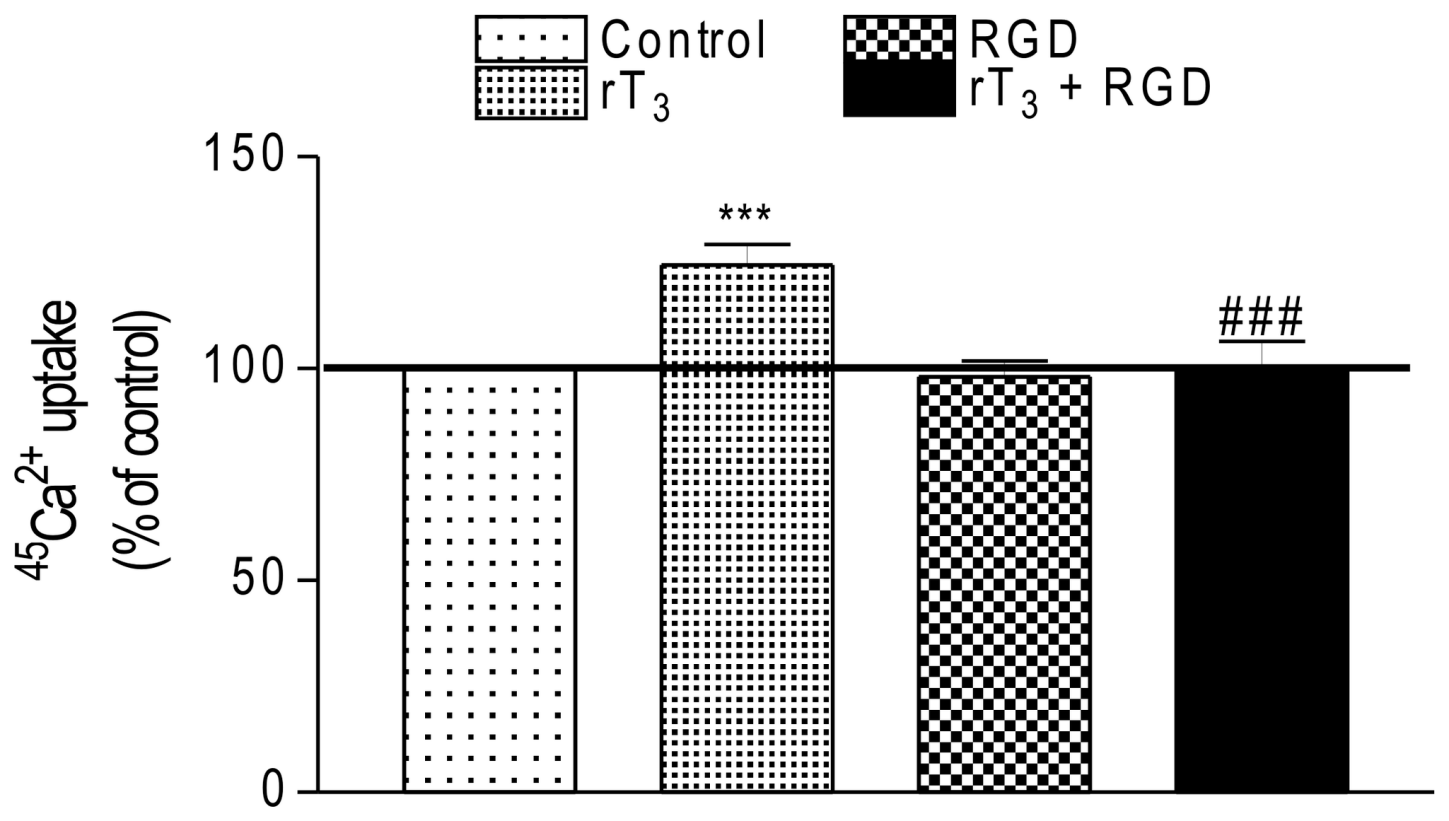
Influence of RGD peptide on stimulatory effect of rT_3_ on ^45^Ca^2+^ uptake in Sertoli cells. Pre-incubation: 15 min in KRb, additional pre-incubation: 60 min with 0.1 µCi/mL of ^45^Ca^2+^ and incubation time: 60 s with 0.1 µCi/mL of ^45^Ca^2+^ in the presence or absence of RGD peptide (5 x 10^-7^ M) with/without rT_3_ (10^-17^ M). Means ± S.E.M. For control, rT_3_, RGD and rT3 + RGD, n=10 for each group. ***p < 0.001 compared with control group; ###*p* < 0.001 compared with rT_3_ group.

In order to evaluate the participation of α_v_β_3_ integrin in the rT_3_ action on ^45^Ca^2+^ uptake, Sertoli cells were exposed to 10^-17^ M of the T_4_ metabolite in the presence or absence of RGD (a peptide that inhibits thyroid hormone binding to integrins) and the ^45^Ca^2+^ uptake was investigated. Figure 2 shows that RGD did not affect the basal calcium uptake. However, the rapid stimulatory effect of rT_3_ on calcium uptake was completely inhibited by the RGD peptide.

### Involvement of voltage-dependent calcium and chloride channels on rT_3_ response in Sertoli cells

**Figure 3:**
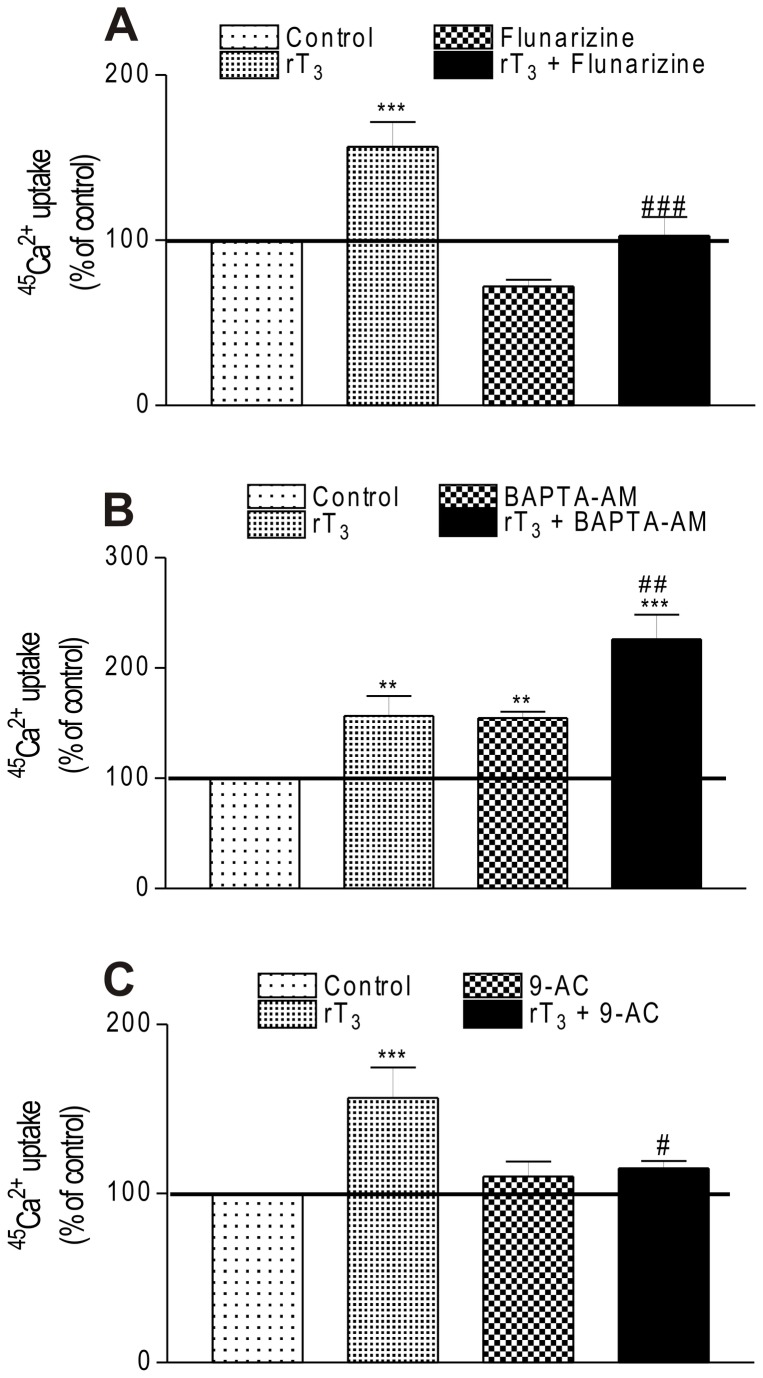
Involvement of ionic channels and intracellular calcium on stimulatory effect of rT_3_ on ^45^Ca^2+^ uptake. (A) Influence of flunarizine, (B) BAPTA-AM and (C) 9-AC on stimulatory effect of rT_3_ on ^45^Ca^2+^ uptake in Sertoli cells. Pre-incubation: 15 min in KRb, additional pre-incubation: 60 min with 0.1 µCi/mL of ^45^Ca^2+^ and incubation time: 60 s with 0.1 µCi/mL of 45Ca2+ in the presence or absence of flunarizine (1 µM), BAPTA-AM (50 µM) and 9-AC (1 µM) with/without rT3 (10-17 M). Means ± S.E.M. For control, n=10; rT3, n=7; flunarizine, n=8; rT3 + flunarizine, n=8; BAPTA-AM, n=8; rT3 + BAPTA-AM, n=6; 9-AC, n=6; rT3 + 9-AC, n=6. ***P < 0.001 and **p < 0.01 compared with control group; ###p < 0.001; ##p < 0.01 and #p < 0.05 compared with rT3 group.

We also investigated whether T-type voltage-dependent calcium channels (T-VDCC) could be involved in the rT_3_ stimulatory action on ^45^Ca^2+^ uptake. To this aim, Sertoli cells were incubated in the presence of rT_3_ with/without flunarizine (1 µM) which blocks T-VDCCs [18]. In Figure 3A it can be observed that flunarizine nullified the rT_3_ stimulatory effect indicating the involvement of T-type VDCC in the calcium uptake in Sertoli cells. 

Once the participation of T-VDCC on ^45^Ca^2+^ uptake in Sertoli cells had been established, we also sought to determine whether intracellular calcium levels could play a role in regulating the VDCC activity by using BAPTA-AM. Our findings demonstrated that when intracellular calcium was chelated by BAPTA-AM the ^45^Ca^2+^ uptake significantly increased and when BAPTA-AM was co-incubated with rT_3_ the stimulatory effect of rT_3_ was potentiated (Figure 3B). 

Since VDCCs can open in response to changes in the resting plasma membrane potential, we investigated whether chloride influx might lead to ^45^Ca^2+^ uptake through VDCCs. The use of a specific blocker for calcium-dependent chloride channels (9-AC) demonstrated that it prevented the rT_3_-induced ^45^Ca^2+^ uptake (Figure 3C). These data allowed us to establish the implication of T-VDCC, intracellular calcium and chloride channels in mediating signal transduction of rT_3_ in immature Sertoli cells. 

### rT_3_ effect on ^45^Ca^2+^ uptake is mediated by PKC and MEK

**Figure 4:**
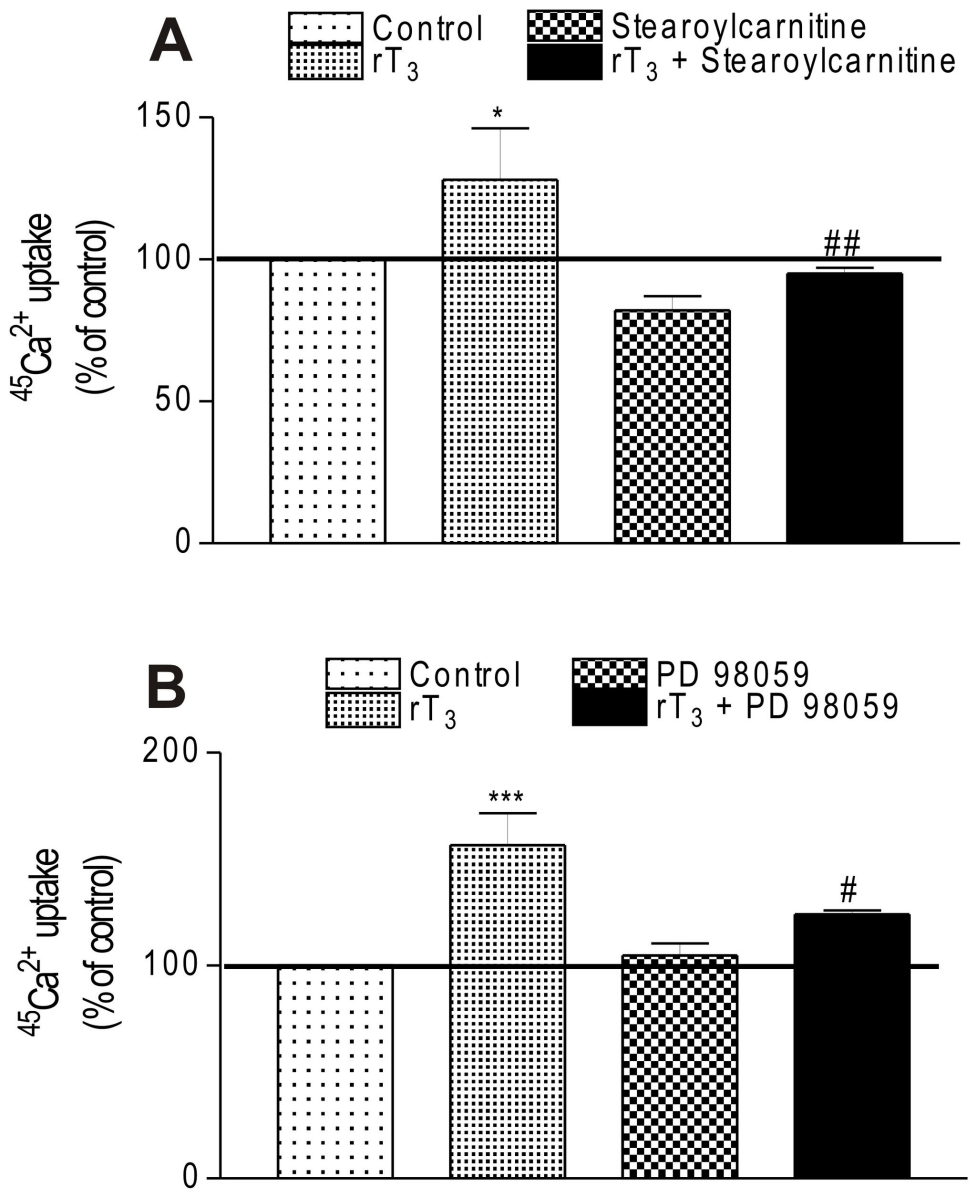
Involvement of kinases proteins on stimulatory effect of rT_3_ on ^45^Ca^2+^ uptake in Sertoli cells. (A) Influence of stearoylcarnitine and (B) PD 98059. Pre-incubation: 15 min in KRb, additional pre-incubation: 60 min with 0.1 µCi/mL of ^45^Ca^2+^ and incubation time: 60 s with 0.1 µCi/mL of ^45^Ca^2+^ in the presence or absence of stearoylcarnitine (1 µM) and PD 98059 (30 µM) with/without rT_3_ (10^-17^ M). Means ± S.E.M. For control, n=9; rT_3_, n=6; stearoylcarnitine, n=8; rT_3_ + stearoylcarnitine, n=9; PD 98059, n=8; rT_3_ + PD 98059, n=8. ***p < 0.001 and *p < 0.05 compared with control group; ##*p* < 0.01 and #*p* < 0.05 compared with rT_3_ group.

The contribution of different protein kinases known to target the calcium channels proteins [39] was investigated. To this aim, stearoyl carnitine and PD 98059 were used as PKC and MEK inhibitors, respectively. As shown in Figure 4, exposure to rT_3_ at 10^-17^ M for 60 s was able to increase the ^45^Ca^2+^ uptake but when the cells were previously preincubated with the kinase inhibitors the stimulatory effect of the hormone was totally prevented.

### rT_3_ and Sertoli cell secretion

**Figure 5:**
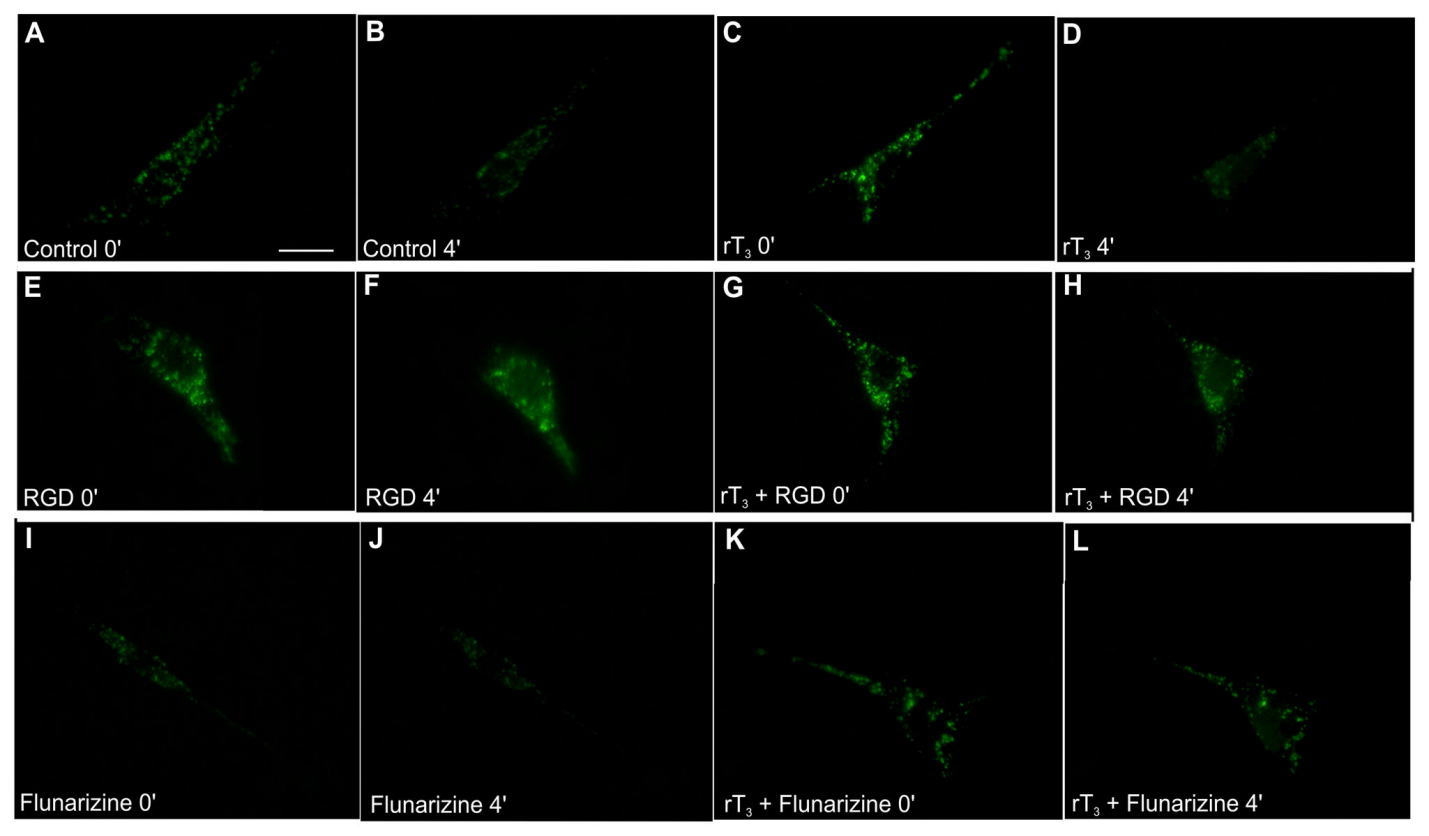
Fluorescence images of Sertoli cells stained with quinacrine. Quinacrine stains individual secretory vesicles in the cell cytoplasm. Sertoli cells in culture were incubated with 3 µM quinacrine for 30 min, washed and photographed under fluorescence illumination immediately (A and C) and at 1 min intervals for 10 min of incubation in the absence or presence of rT_3_, respectively (B and D). Incubation of cells with 10^-17^ M rT_3_ caused fusion of quinacrine-loaded vesicles to the plasma membrane and release of the fluorescent content into the surrounding medium, as seen by the loss of fluorescence from most vesicles located at the cell periphery. This effect was observed after 4 min incubation with rT_3_. Also, Sertoli cells were incubated for 10 min with 500 nM of RGD peptide or 1 µM of flunarizine prior to incubation with quinacrine, washed and incubated with 3 µM quinacrine for 30 min. Quinacrine-loaded Sertoli cell cultures, pre-treated with RGD or flunarizine for 10 min, were incubated in the absence or presence of 10^-17^ M rT_3_ and photographed under fluorescence illumination immediately (E, G, I and K) and at 1-min intervals for 10 min of incubation in the absence or presence of rT_3_ (F, H, J and L). Incubation of cells in the presence of 500 nM RGD peptide or 1 µM flunarizine prevented the fusion of quinacrine-loaded vesicles to the plasma membrane and release of the fluorescent content. (A) Control, 0 min. (B) Control, 4 min. (C) rT_3_, 0 min. (D) rT_3_, 4 min. (E) RGD, 0 min. (F) RGD, 4 min. (G) rT_3_ + RGD, 0 min. (H) rT_3_ + RGD, 4 min. (I) Flunarizine, 0 min. (F) Flunarizine, 4 min. (G) rT_3_ + Flunarizine, 0 min. (H) rT_3_ + Flunarizine, 4 min. Experiments were performed 3 times with similar results. Bar = 10 µm.

To demonstrate the potential of rT_3_ to induce cellular secretion, Sertoli cells were labeled with quinacrine. The panel in Figure 5 shows quinacrine loading in Sertoli cells monitored by fluorescence microscopy through changes in the fluorescence intensity or fluorescence lifetime. The images revealed non-uniform quinacrine distribution within the cells with punctate staining, which results from vesicular accumulation of the dye. In Figure 5A the granular staining is evident in the cytoplasm, but it was particularly abundant in the perinuclear region. Figure 5B represents a single quinacrine-stained cell imaged after 4 min in the basal condition without significant changes in fluorescence intensity. To study the exocytosis of quinacrine-stained vesicles induced by rT_3_, fluorescence changes of individual cells were analyzed and compared with basal conditions. Comparing the image in Figure 5D with the control (Figure 5C) demonstrates that after 4 min of exposure to rT_3_ the cells manifested a loss of fluorescence indicating an exocytic quinacrine release.

Since the results for the calcium uptake indicated rapid response and plasma membrane-associated rT_3_ actions, we investigated the participation of α_v_β_3_ integrin and T-type VDCCs in the mechanism of action of rT_3_ in Sertoli cell secretion. The findings demonstrated that RGD peptide and flunarizine did not produce alterations in cellular secretion (Figure 5F and 5J) when compared with respective control cells (Figure 5E and 5I). In addition these blockers prevented the exocytosis induced by rT_3_ (Figure 5H and 5L).

## Discussion

Thyroid hormones T_3_ and T_4_ give rise to a wide range of effects on metabolism, growth and development [43]. T_4_ is the major form of TH secreted by the thyroid gland, whereas T_3_ is produced mainly in target tissues by deiodination of T_4_ [44]. While it is clear that many of the thyroid hormone actions are mediated by T_3_-dependent regulation of gene expression, in recent years the nongenomic action of thyroid hormones has also been reported (for review see [45]). Particularly in the male reproductive system, thyroid hormones play an important role where they regulate a diverse set of functions through rapid and genomic mechanisms (for review see [46]). 

In this study, we obtained novel evidence that rT_3_, a T_4_ metabolite until recently regarded as inactive, is also involved in the regulation of 11 day-old Sertoli cell functions. It was demonstrated that rT_3_ stimulates calcium uptake in these cells within a very short time (60 s) and with a very low concentration (10^-17^ M) compared to thyroxine. A similar effect was previously reported by our group for T_4_ in whole testis [14]. However, the minimum concentration of T_4_ required to induce calcium influx in the testis was 10^-9^ M, highlighting the greater potency of rT_3_ when compared to T_4_. 

Although the enzymes deiodinase 1 (D1) and deiodinase 3 (D3), which inactivate T_4_ and T_3_ by converting them to their reverse T_3_ (rT_3_) and 3,3′-T_2_ forms, respectively, exist in prepubertal and pubertal rat testis [3,47], there are no reports concerning the effect of rT_3_ in the testis or in Sertoli cells. Thus, as far as we are aware, this is the first demonstration of the rapid response of rat Sertoli cells in relation to calcium uptake by rT_3_. Based on this finding, the very potent effect of rT_3_ observed herein may represent a cell-specific modulatory event independent of high amounts of TH metabolites produced by the liver [48,49].

Nevertheless, several questions remain unanswered concerning the TH mechanism of action in the male reproductive system, especially related to rapid and nongenomic effects. For many years, TH action was viewed as dependent on the presence of nuclear receptors (TRs) and their major ligand, T_3_. Identification of a cell surface receptor for TH provides a molecular basis for certain nongenomic effects. Plasma membrane integrin α_v_β_3_ is a cell surface receptor described for TH in the central nervous system, where nongenomic actions are initiated. It has been shown that integrin α_v_β_3_ contains a binding domain for iodothyronines [50]. This domain contains an Arg-Gly-Asp (RGD) recognition site that is important for the binding of a variety of extracellular proteins and growth factors [51,52]. A family of adhesion proteins known as integrins has been described in relation to the reproductive system [53]. In addition, α_6_β_1_ integrin is expressed in Sertoli cells involved in cell-cell junctions [54]. In this regard, the RGD peptide was used to determine whether rT_3_-induced calcium uptake is mediated by integrin and, as expected, the results confirmed the participation of integrin in rT_3_ action on Sertoli cells.

Calcium helps regulate a variety of cellular functions in different cells, including germ cells and somatic cells in the testis in response to hormones and local regulators [55]. Considering the relevance of calcium overload on the modulation of a variety of Sertoli cell functions, especially cell secretion, different channel blockers and kinase inhibitors were used to determine the role and the mechanism of action of rT_3_ in calcium uptake. The rapid and/or sustained calcium uptake through VDCC seems to be required for physiological responses in Sertoli cells [14,16]. Therefore, in order to clarify its involvement in rT_3_ action, the T-VDCCs were previously inhibited with the use of a known calcium channel blocker. Our results showed that flunarizine totally prevented the rT_3_ effect on calcium uptake as has been reported for other hormones, such as T_4_, T_3_ and 1,25(OH)_2_ vitamin D_3_ [18,56]. Zamoner et al. [56] have demonstrated that the effects of T_4_ and T_3_ on the cerebral cortex of young rats are mediated by both L- and T-type VDCCs. Likewise, Rosso et al. [18] recently showed the involvement of T-type VDCC in calcium uptake induced by 1,25(OH)_2_ vitamin D_3_ in 10-day-old rat testis. These findings demonstrate that rT_3_-induced calcium uptake was directly and mostly related to VDCC.

The entrance of calcium into Sertoli cells can be triggered by depolarization, channel protein phosphorylation or depletion of intracellular calcium stores which requires functioning VDCC [33]. Electrophysiological studies demonstrated that T-type calcium channels of excitable cells are located in the plasma membrane of immature Sertoli cells [27]. Our findings indicated that calcium uptake induced by rT_3_ can result from T-VDCC opening but not from intracellular calcium depletion, since co-incubation of BAPTA-AM and rT_3_ produced a significant increase in calcium influx compared with that produced by rT_3_ alone. In order to evaluate the mechanisms that could lead to calcium uptake through T-VDCC in Sertoli cells, we investigated the participation of ionic channels and protein kinases by using pharmacological tools which allowed us to determine that effect of rT_3_ on calcium uptake is dependent on the chloride channel as well as PKC and MEK. In this context, the calcium influx through T-VDCCs could be modulated by complex mechanisms involving the activities of these protein kinases [46,57] or by changes in the plasma membrane potential generated by the opening of ionic channels [58].

Several hormones which regulate T-VDCCs have the ability to conduct calcium across the cellular membrane at potentials close to the resting potential [57]. In the testis, modulation of the voltage-dependent calcium conductance by changing the chloride concentration has been described [28]. Also, we recently showed the nongenomic effect of 1,25(OH)_2_ vitamin D_3_-induced calcium uptake in Sertoli cells through L- and T-VDCC modulation by Ca^2+^-dependent chloride channels [17,18] as well as in the cerebral cortex of young rats [59]. Also, T-channel activity, like that of most ion channels, can be modulated by hormones acting through signaling pathways such as protein kinases A and C [42]. 

It has been reported that PKC can modulate T-VDCC in a variety of cell systems [57]. Herein, we reveal the involvement of PKC in the calcium influx via T-VDCC in Sertoli cells. Besides the stimulatory effect of 1,25(OH)_2_-vitamin D_3_ on calcium uptake in the testis or Sertoli cells mediated by PKC and PKA, Costa et al. [60] also reported that the luteinizing hormone (LH) modulates T-type calcium currents in Leydig cells through PKA and PKC. 

It has been reported that both conventional and novel PKCs can activate the MAPK signaling pathway [61] and, therefore, we also investigated the involvement of MEK in rT_3_-induced calcium uptake. The participation of MEK in calcium influx demonstrated in this study is in agreement with previous reports by our group for the effect of 1,25(OH)_2_-vitamin D_3_ on testis [17] and Sertoli cells [18].

There is an increasing body of evidence that T-type calcium channels can trigger fast and low-threshold exocytosis in neurons [62] as well as in chromaffin cells [63] and in retinal glial cells [64] controlling the release of neurotransmitters. In addition, these authors have reported that these channels are equally distributed near the docked secretory vesicles [63].

In this context, this study adds important evidence demonstrating that exocytosis in immature rat Sertoli cells is modulated by rT_3_. Similar granular quinacrine staining has been reported in the mouse Sertoli cell line (TM4) treated with 1,25(OH)_2_-vitamin D_3_, which was related to chloride channel activation [42]. Herein, the results reported suggest the involvement of calcium channels in cellular secretion induced by rT_3_. Moreover, the data obtained indicate that exocytosis is mediated by integrin and T-type VDCCs, since the preincubation of the cells with RGD and flunarizine abrogated the fusion of fluorescent vesicles with the plasma membrane leading to the disappearance of fluorescence.

Collectively, our findings reveal a new active metabolite of thyroid hormone in immature Sertoli cell. Our results strongly suggest that rT_3_ increases the calcium influx and that T-type VDCCs activation is implicated in Sertoli cell secretion. The activity of T-type VDCCs could be regulated by rT_3_ through integrin binding and consequent PKC, MEK and chloride channel activation. The modulation of calcium entry into Sertoli cells by rT_3_ might participate in the regulation of intracellular processes, such as cell secretion, reinforcing the role of rT_3_ in the male reproductive system physiology. Future studies are necessary to analyze further the physiological relevance of rT_3_ as well as to characterize the specific types of integrin that preferentially bind to the hormone in Sertoli cells. Ultimately, such knowledge could lead to the identification of novel means to regulate these possible physiological actions for therapeutic purposes.
